# Bibliometric analysis of research on immunogenic cell death in cancer

**DOI:** 10.3389/fphar.2022.1029020

**Published:** 2022-10-06

**Authors:** Yan Zhou, Fen Hu, Yang Cui, Haiyang Wu, Shunan Hu, Wei Wei

**Affiliations:** ^1^ Clinical College of Neurology, Neurosurgery and Neurorehabilitation, Tianjin Medical University, Tianjin, China; ^2^ Department of Oncology, XiangYang Central Hospital, Hubei University of Arts and Science, XiangYang, China; ^3^ Institute of Oncology, XiangYang Central Hospital, Hubei University of Arts and Science, XiangYang, China; ^4^ Department of Neurosurgery, Hebei Yanda Hospital, Langfang, China; ^5^ Department of Neurosurgery, XiangYang Central Hospital, Hubei University of Arts and Science, XiangYang, China

**Keywords:** immunogenic cell death, cancer immunotherapy, bibliometric analysis, tumor microenvironment, nanotechnology

## Abstract

**Background:** Immunotherapy is changing the way we treat cancer. Immunogenic cell death (ICD) has received considerable attention in the treatments of various cancer types, due to the long-lasting antitumor responses elicited in human body. However, to date, no relevant bibliometric research has been reported.

**Methods:** Publications related to ICD in cancer research were collected from the Web of Science Core Collection. Using CiteSpace, VOSviewer and an online platform, the analyses of co-author, co-citation, and co-occurrence of terms retrieved from literatures were carried out.

**Results:** A total of 1,577 publications were included in this study. The global research literatures on ICD in cancer research have been increasing from 2005 to 2021. China, the United States and France dominated in this area and had close collaborations with many countries. Six of the top 10 most contributive institutions were from France. When it comes to author analysis, Kroemer G, Zitvogel L, Kepp O, Garg AD and Galluzzi L were in both the top 10 most productive authors and top 10 most co-cited authors lists. The co-occurring author keywords could be grouped into three clusters: “biomarkers of ICD”, “nanoparticles” and “combination therapy”. In terms of promising hotspots, keywords (author keywords and KeyWords Plus) with recent citation bursts could be summarized into two aspects: “tumor microenvironment” and “nanoparticles”.

**Conclusion:** Increased attention has been paid to ICD in cancer treatment. However, there are still many unresolved domains in the field of ICD, such as clinical application and molecular mechanisms of this cell death process. ICD-inducing modalities combined with nanotechnology could potentiate the current immunotherapies, and will be hotspots for future research.

## Introduction

An innovative paradigm for treating cancer has been unlocked by finding mechanisms that break down the immunity-cancer barrier. Immunotherapies, such as chimeric antigen receptor T (CAR T) cells, tumor vaccines and immune checkpoint inhibitors (ICIs), have shown great promise in treating cancer, according to a series of recent clinical trials (Weber et al., 2015; Li et al., 2016; June et al., 2018). It is worth noting, however, that most of these frequently used immunotherapies are ineffective when applied to certain cancers, such as glioblastoma, which exhibits high heterogeneity and a “cold state” of the immune microenvironment (Gedeon et al., 2020; Reardon et al., 2020).

In the past two decades, the concept of immunogenic cell death (ICD) has emerged, that is, a form of regulated cell death (RCD) that can stimulate immune responses against dying/dead-cell antigens, especially those derived from tumor cells (Kroemer et al., 2013; Fucikova et al., 2020). This concept was first proposed during anticancer chemotherapy, when adaptive immune responses against residual cancer cells can determine the effectiveness of conventional chemotherapeutic agents (Zitvogel et al., 2011). Damage-associated molecular patterns (DAMPs) refer to a group of molecules released by dying or damaged cells or tissues, like adenosine triphosphate (ATP) and calreticulin (CRT), which can either act as adjuvants or warning signals for the immune system (Krysko et al., 2012). During the process of ICD, reactive oxygen species (ROS) and endoplasmic reticulum stress (ERS) are two major factors in the production of DAMPs (Kepp et al., 2015; Li et al., 2019). Nevertheless, the process of this cell death modality remains an enigma because the underlying molecular mechanisms are not fully understood. In addition to chemotherapeutic agents, oncolytic viruses, photodynamic therapy (PDT), and radiotherapy also have the potential to cause ICD (Zhou et al., 2019). Thus, induction of ICD in tumor cells is well-suited for provoking long-term efficacy of antitumor treatments, combining direct cancer cell killing and antitumor immune responses.

Today, bibliometric analysis is widely used to determine the quality, scholarly impact, and centrality of a body of literature within a particular scientific field, using mathematical and statistical approaches (Synnestvedt et al., 2005; Chen and Song, 2019; Wu et al., 2021a). Meanwhile, it is also recommended as a way to find hotspots, research frontiers, or emerging trends in academic fields by evaluating metrological characteristics of publications, including quantity of publications, collaboration and citation/co-citation frequency, academic journals, references and keywords. In the field of biomedical, a plenty of bibliometric analyses have been performed to improve the understanding of specific research domains (Merigo and Nunez, 2016; Wu et al., 2021b).

Over the past few decades, there have been a growing number of studies focused on ICD. However, to our knowledge, there exists no systematic evaluation of the published literature on this topic. Herein, we present an overview of the ICD field in cancer, and identify new directions for inducing ICD in cancer treatment, in light of fact that ICD studies are predominantly focused on malignant diseases.

## Materials and methods

### Dataset selection

All literatures on ICD in cancer were searched based on the Science Citation Index Expanded of Web of Science Core Database (WOSCC) (https://www.webofscience.com), which is the most-acknowledged database in bibliometric analyses (Vanzetto and Thome, 2019; Usman and Ho, 2020). The literatures between 2005 and 2021 regarding ICD and cancer were retrieved on 2 May 2022. We searched “immunogenic cell death” and “cancer” and their synonyms in the title/abstract/author keywords of literatures, and the detailed searching strategy was shown in the Supplementary file. Only “articles” and “review articles” were considered.

### Data visualization and analysis

To determine the significance of a publication, we assumed that highly cited publications had greater impacts on a research domain than those that received fewer citations. Co-citation occurs when two related items (authors or articles) are cited by a third item (author or article) at the same time. The annual global publications and citations was visualized by WoS web tool. The collaboration networks between countries or regions were constructed by an online platform for bibliometric analysis (http://bibliometric.com). VOSviewer (version 1.6.16) was used to visualize the networks between countries/regions, institutions researchers and keywords, and achieve co-authorship, citation, co-citation, and co-occurrence analyses (van Eck and Waltman, 2010). In VOSviewer, nodes represent different terms, and the links between nodes showed the correlations between terms, which were quantitatively evaluated by total link strength (TLS). Meanwhile, cluster and timeline of co-citation references and dual-map overlay of journals were completed by CiteSpace (version 5.8.R3) (Synnestvedt et al., 2005). Additionally, keywords and references with citation bursts were also determined by CiteSpace. The setting parameters of CiteSpace were as follows. From 2005 to 2021, one or 2 years per slice was set for time slicing. For burst detection, top 50 from each time slice was set for references while top 60 was set for keywords. Pathfinder and pruning sliced networks were chose for pruning. England, Scotland, Northern Ireland, and Wales were grouped as the United Kingdom (UK).

## Results

### Trend of global publications and citations

**FIGURE 1:**
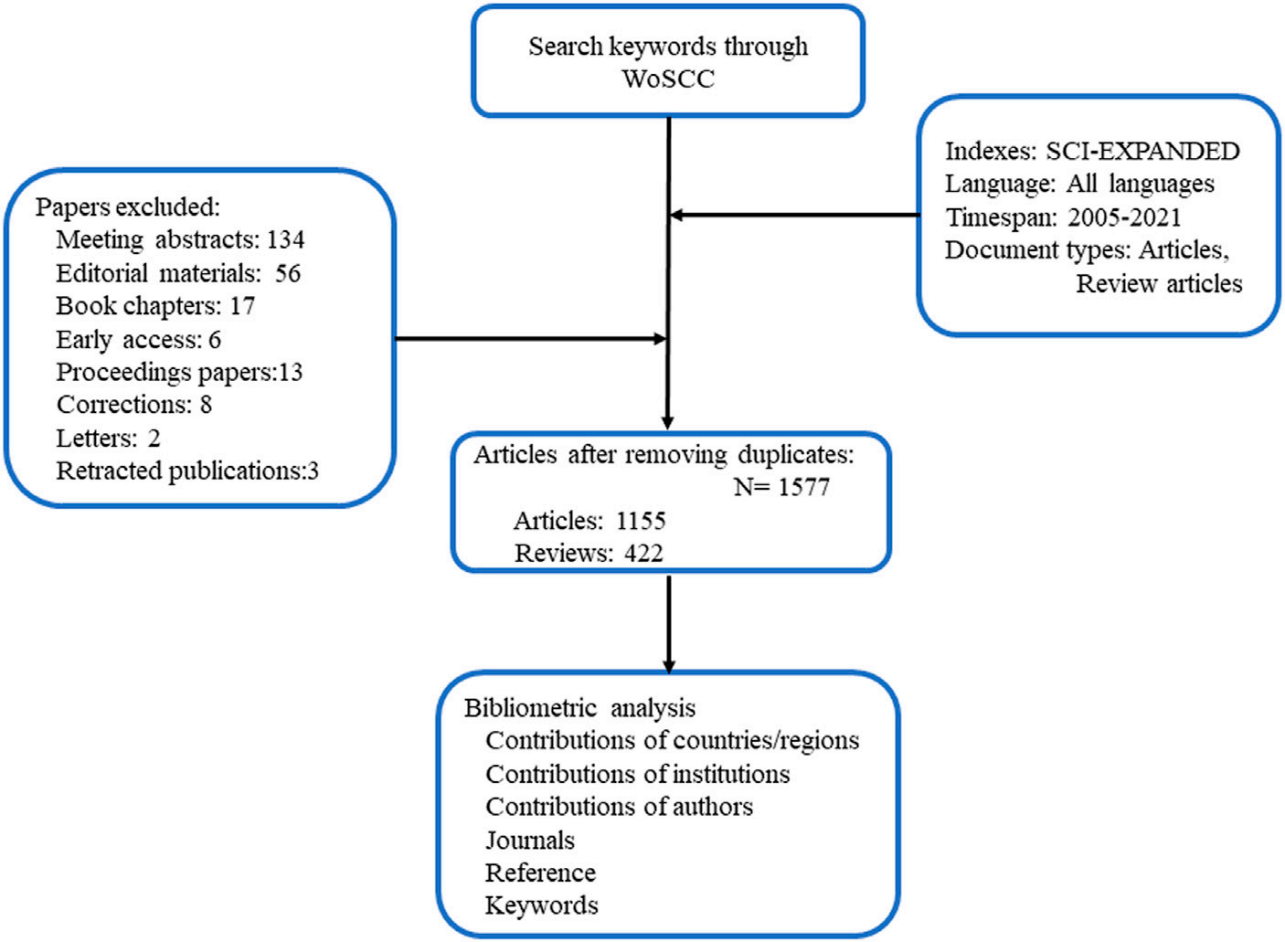
Schematic diagram of the search process.

**FIGURE 2:**
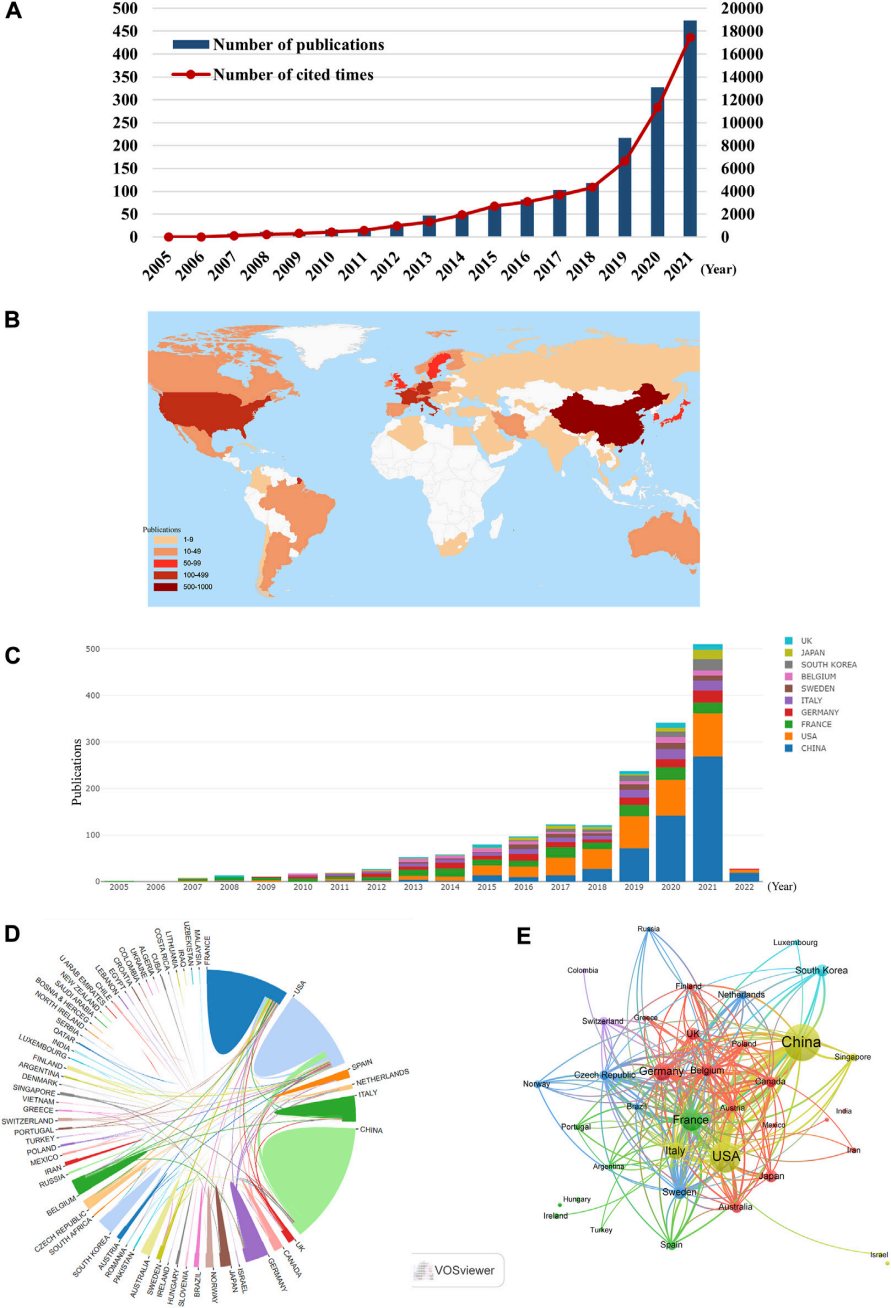
**(A)** The annual number and total citations of articles related to ICD in cancer research from 2005 to 2021. **(B)** World map based on the number of articles of countries/regions. **(C)** The trend of annual number of articles from top 10 countries/regions. **(D)** The international collaborations between countries/regions. The links between countries/regions represented the frequency of the collaborations. **(E)** The citation network of countries/regions, conducted by VOSviewer. The size of nodes indicated the number of articles, while the thickness of links indicated the citation strength.

From 2005 to 2021, 1,577 publications including 1,155 articles and 422 reviews were collected from the WoSCC (Figure 1). Most of publications were published in English (1,568), followed by French (Gedeon et al., 2020), German (Weber et al., 2015) Spanish (Li et al., 2016), and Chinese (Li et al., 2016). The research on ICD in cancer has steadily increased over the last few decades [from 1 (2005) to 474 (2021)], with a noticeable rise over the past 3 years (Figure 2A). The papers received an average of 38.95 citations per paper, for a total of 61,419 citations.

### Countries/regions and institutions analysis

According to the world map, 61 countries/regions have published articles on this topic, with China (576), the United States (401) and France (202) contributing the most (Figures 2B,C, Table 1). Moreover, France and the United States received higher citations than China (Table 1). An interactive cooperation map plots the cooperation relationships between countries/regions (Figure 2D). In spite of producing more papers than the United States and France, China did not collaborate with other countries/regions as frequently as the United States and France. In terms of citation analysis, the citation relationships between the 38 countries/regions which published at least five documents were visualized in Figure 2E.

**TABLE 1:** Top 10 productive countries/regions related to ICD in cancer research.

Rank	Countries/regions	Documents (N)	Percentage (N/1,577)	Citations	Citations per paper
1	China	576	36.53	12170	21.13
2	United States	401	25.43	18759	46.78
3	France	202	12.81	19483	96.45
4	Germany	134	8.50	5,413	40.40
5	Italy	119	7.55	7,289	61.25
6	Sweden	72	4.57	4,735	65.76
7	Belgium	69	4.38	6,477	93.87
8	South Korea	62	3.93	1,099	17.73
9	Japan	60	3.80	1730	28.83
10	United Kingdom	57	3.61	2,733	47.95

**FIGURE 3:**
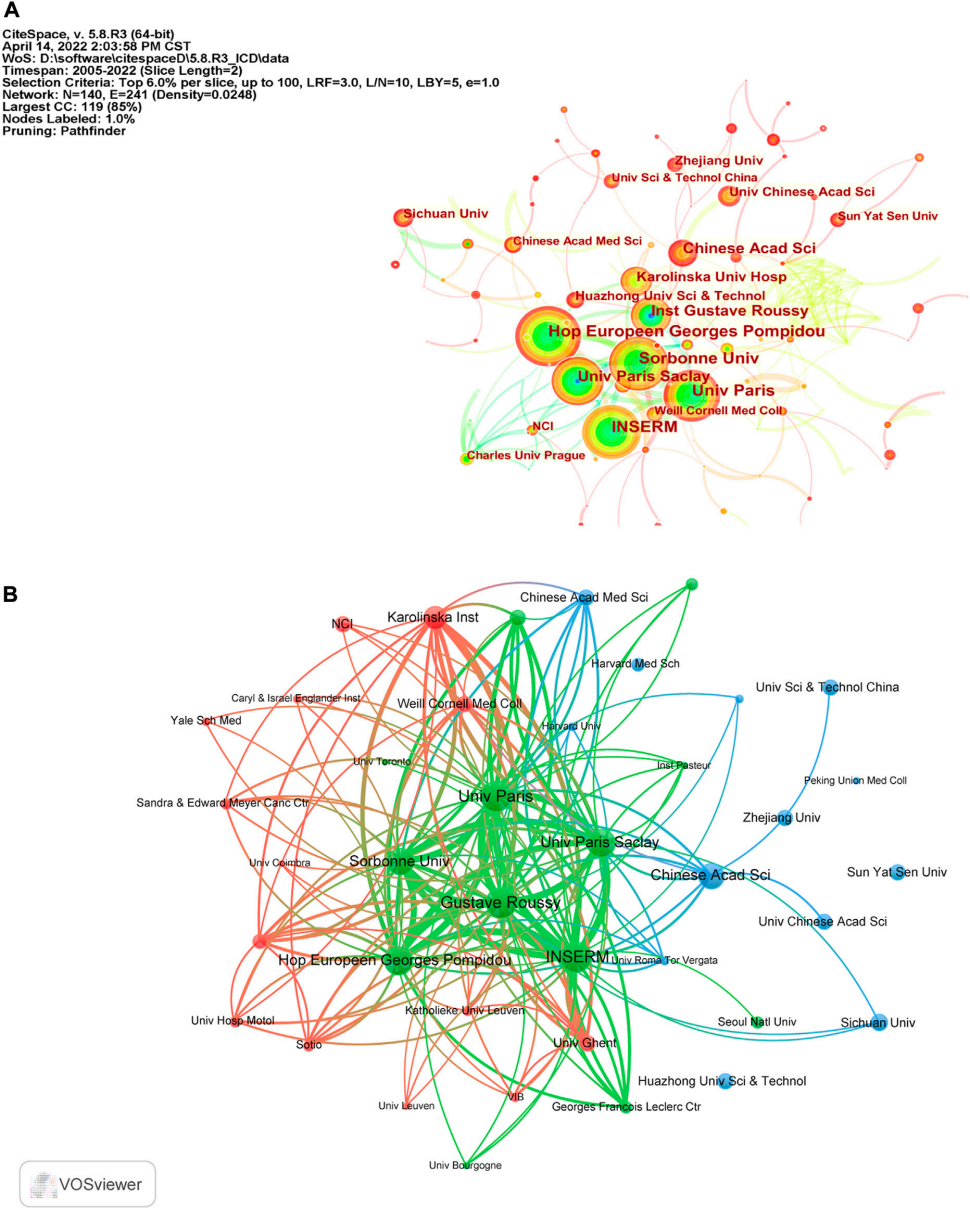
**(A)** The cooperation map of institutions performed with CiteSpace. The size of nodes represented the publication counts, while the thickness of the lines indicated the collaboration strength. **(B)** The citation network of institutions generated by VOSviewer.

According to CiteSpace, 1,577 documents were contributed by 1,815 different institutions. The top 10 institutions with the most articles are listed in Table 2. And it could be found that France occupied the top six of the 10 institutions with the greatest number of articles, which may be attributed by the frequent cooperation among these institutions (Figure 3A). It was also discovered that institutions from the United States did not appear in the top 10 list of postings. When it comes to citation analysis of institutions, the top three institutions with the largest citations were Gustave Roussy (15331), INSERM (14765) and Univ Paris Saclay (14455) (Table 2, Figure 3B).

**TABLE 2:** Top 10 institutions ranked by the numbers of publications.

Rank	Institutions	Documents (N)	Citations	TLS	Countries/regions
1	Gustave Roussy	107	15331	19554	France
2	INSERM	107	14765	19685	France
3	Univ Paris	107	12438	18779	France
4	Univ Paris Saclay	91	14455	18174	France
5	Hop Europeen georges pompidou	90	8,887	15195	France
6	Sorbonne univ	86	8,871	14505	France
7	Chinese acad sci	76	2,301	3,985	China
8	Karolinska univ hosp	41	2,297	5,108	Sweden
9	Sichuan univ	35	498	1,017	China
10	Zhejiang univ	32	519	989	China

INSERM, Institut National de la Santé et de la Recherche Medicale. TLS, total link strength.

### Authors and co-cited authors

**FIGURE 4:**
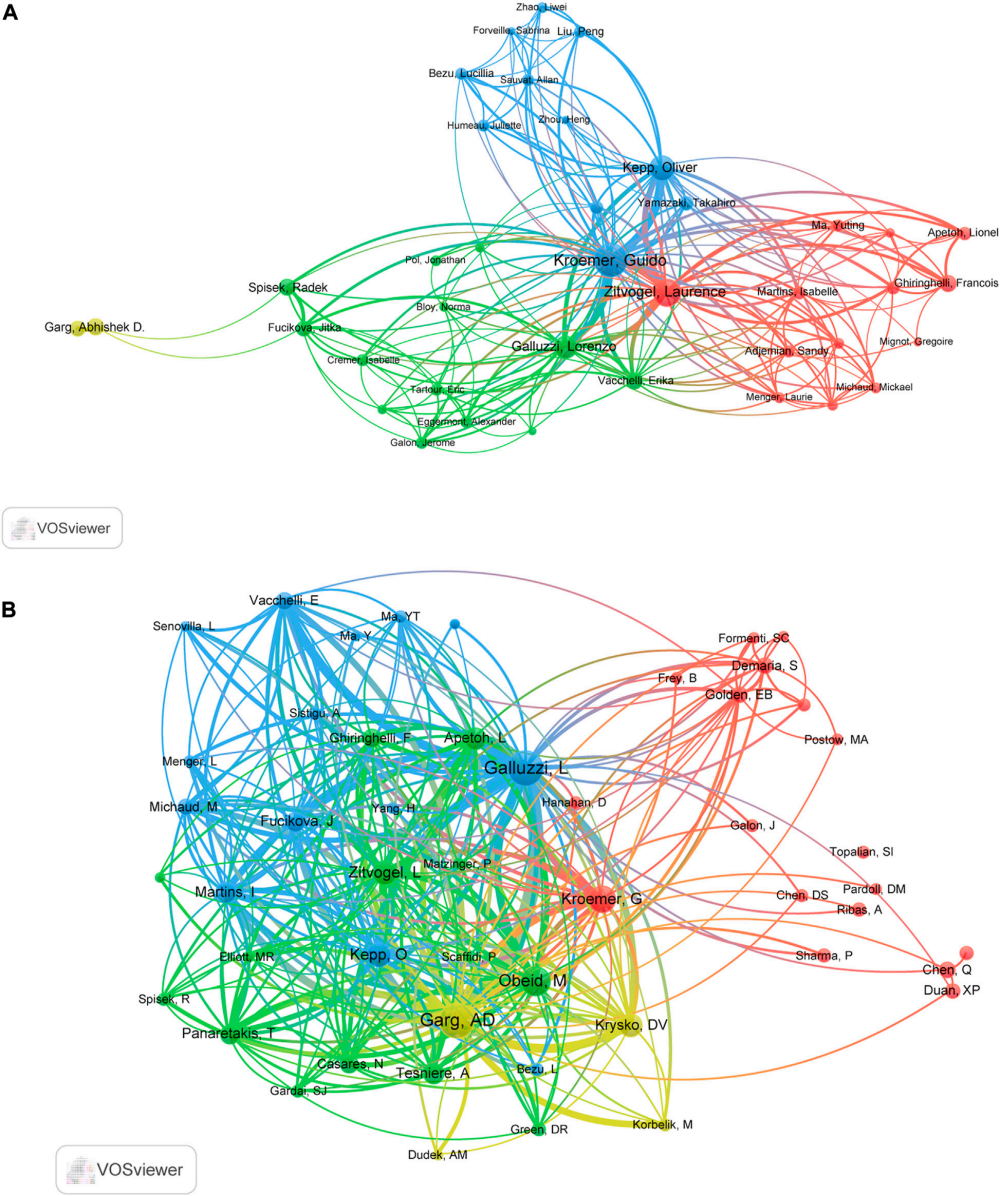
The cooperation map of authors **(A)** and co-citation map of authors **(B)** performed with VOSviewer.

In total, 8,769 authors have contributed to the research on ICD in cancer. The top 10 prolific authors and top 10 co-cited authors with largest TLS were are listed in Table 3. With 94, 64, and 51 papers, Kroemer G, Zitvogel L, and Kepp O were the top three prolific authors (Table 3). As for the co-citation analysis, the top 50 authors with the largest TLS were grouped into four main clusters as seen in Figure 4B. Garg AD had the largest TLS (54116), followed by Galluzzi L (TLS = 48240) and Obeid M (TLS = 25956) (Table 3).

**TABLE 3:** Top 10 authors and co-cited authors in the field of ICD in cancer research.

Rank	Author	Documents	Citations	TLS	Co-cited author	Citations	TLS
1	Kroemer G	94	12733	605	Garg AD	1,112	54116
2	Zitvogel L	64	11700	471	Galluzzi L	1,103	48240
3	Kepp O	51	7,173	368	Obeid M	768	25956
4	Galluzzi L	40	6,701	285	Kroemer G	624	21275
5	Garg AD	22	3,608	70	Zitvogel L	558	23565
6	Spisek R	22	1,699	130	Kepp O	533	20269
7	Ghiringhelli F	20	4,769	134	Krysko DV	497	20028
8	Vacchelli E	20	2,152	180	Apetoh, L	452	18318
9	Agostinis P	19	3,509	58	Tesniere A	389	14259
10	Fucikova J	19	1,506	140	Martins I	383	17898

### Journal analysis

There were 431 journals that covered papers on this topic, including 68 journals that published at least five. In Table 4, a total of 385 articles covered in the top 10 prolific journals, representing 24.41% of all articles. All top 10 journals had fairly high academic rankings and almost all of them were in Q1 based on 2021 Journal Citation Reports (JCR). Five had impact factor (IF) greater than 10. *Oncoimmunology* contributed the most articles (*n* = 83), and *Advanced Materials* was the journal with highest IF (32.086).

**TABLE 4:** Top 10 journals related to the research on ICD in tumor.

Rank	Journal title	Documents (N)	Citations	Percentage (N/1,577)	If (2021)	JCR (2021)	Country
1	*Oncoimmunology*	83	3,248	5.26	7.723	Q1	United States
2	*Cancers*	54	399	3.42	6.575	Q1	Switzerland
3	*Frontiers in Immunology*	42	1,561	2.66	8.786	Q1	Switzerland
4	*Biomaterials*	41	917	2.60	15.304	Q1	Netherlands
5	*Journal for Immunotherapy of Cancer*	32	909	2.03	12.469	Q1	United Kingdom
6	*Frontiers in Oncology*	30	467	1.90	5.738	Q2	Switzerland
7	*ACS Nano*	28	1,363	1.78	18.027	Q1	United States
8	*ACS Applied Materials and Interfaces*	25	345	1.59	10.383	Q1	United States
9	*Advanced Materials*	25	1,611	1.59	32.086	Q1	Germany
10	*Cancer Immunology Immunotherapy*	25	676	1.59	6.63	Q1/Q2	United States

IF, impact factor; JCR, journal citation reports.

**FIGURE 5:**
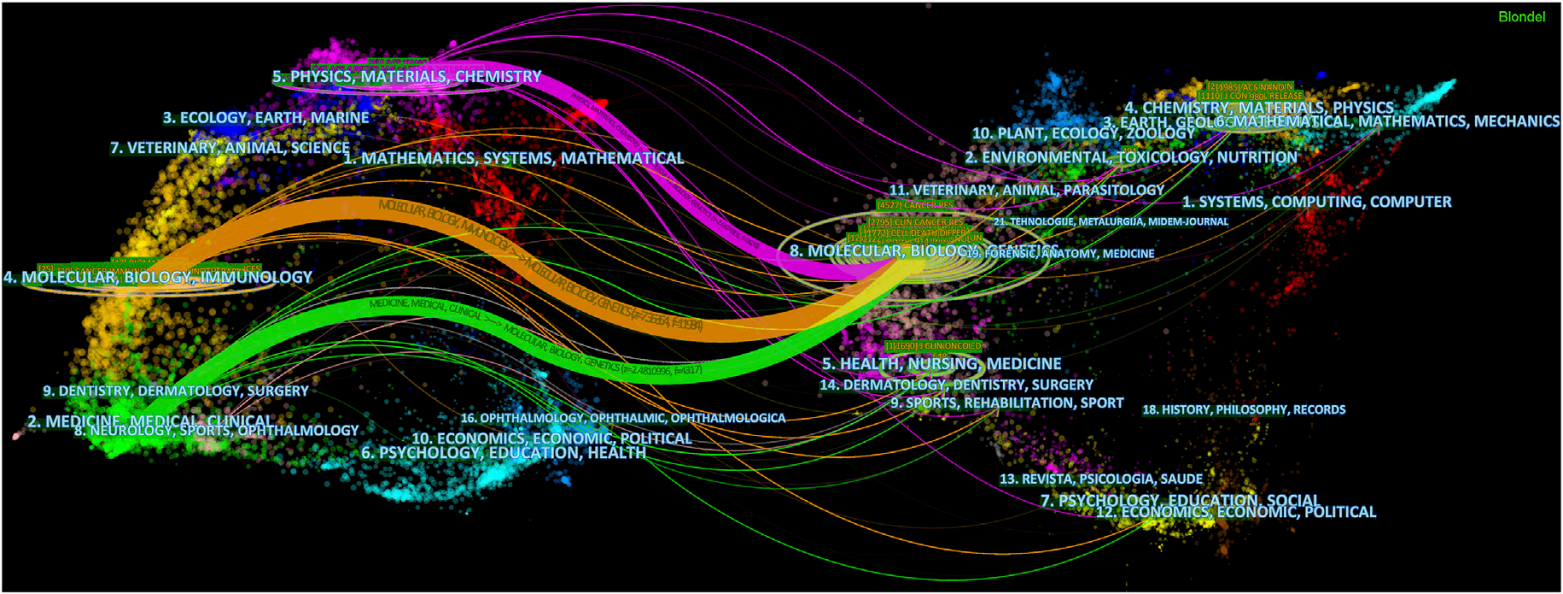
A dual-map overlap of journals on ICD in cancer research created by CiteSpace.

The dual-map overlap of journals illustrates the citation paths between citing journals and cited journals, as Figure 5 denoted, starting from citing journals (left half) and ending with cited journals (right half). The subjects that journals covered were labeled. It could be seen that articles on the subjects of Molecular, Biology and Genetics were most frequently cited, forming three main citation paths that started with “Physics, Materials and Chemistry”, “Molecular, Biology and Immunology” and “Medicine, Medical and Clinical”.

### Analysis of co-cited references

In total, 60825 references were cited by articles on this topic. As shown in Table 5, the article entitled “Calreticulin exposure dictates the immunogenicity of cancer cell death” by Obeid M (Obeid et al., 2007a) had the most co-citations (n = 547). Kroemer G’ paper “Immunogenic cell death in cancer therapy” (Kroemer et al., 2013) ranked the second with 474 co-citations.

**TABLE 5:** The top 10 co-cited references involved in ICD in cancer research.

Title	First author	Journal	Year	Citations
Calreticulin exposure dictates the immunogenicity of cancer cell death	Obeid M	Nature medicine	2007	547
Immunogenic cell death in cancer therapy	Kroemer G	Annual review of immunology	2013	474
Immunogenic cell death and DAMPs in cancer therapy	Krysko DV	Nature reviews cancer	2012	402
Immunogenic cell death in cancer and infectious disease	Galluzzi L	Nature reviews immunology	2017	344
Toll-like receptor 4-dependent contribution of the immune system to anticancer chemotherapy and radiotherapy	Apetoh L	Nature medicine	2007	314
Caspase-dependent immunogenicity of doxorubicin-induced tumor cell death	Casares N	The journal of experimental medicine	2005	289
Mechanisms of pre-apoptotic calreticulin exposure in immunogenic cell death	Panaretakis T	The EMBO journal	2009	247
Immunogenic death of colon cancer cells treated with oxaliplatin	Tesniere A	Oncogene	2010	242
Consensus guidelines for the detection of immunogenic cell death	Kepp O	Oncoimmunology	2014	222
Activation of the NLRP3 inflammasome in dendritic cells induces IL-1beta-dependent adaptive immunity against tumors	Ghiringhelli F	Nature medicine	2009	213

**FIGURE 6:**
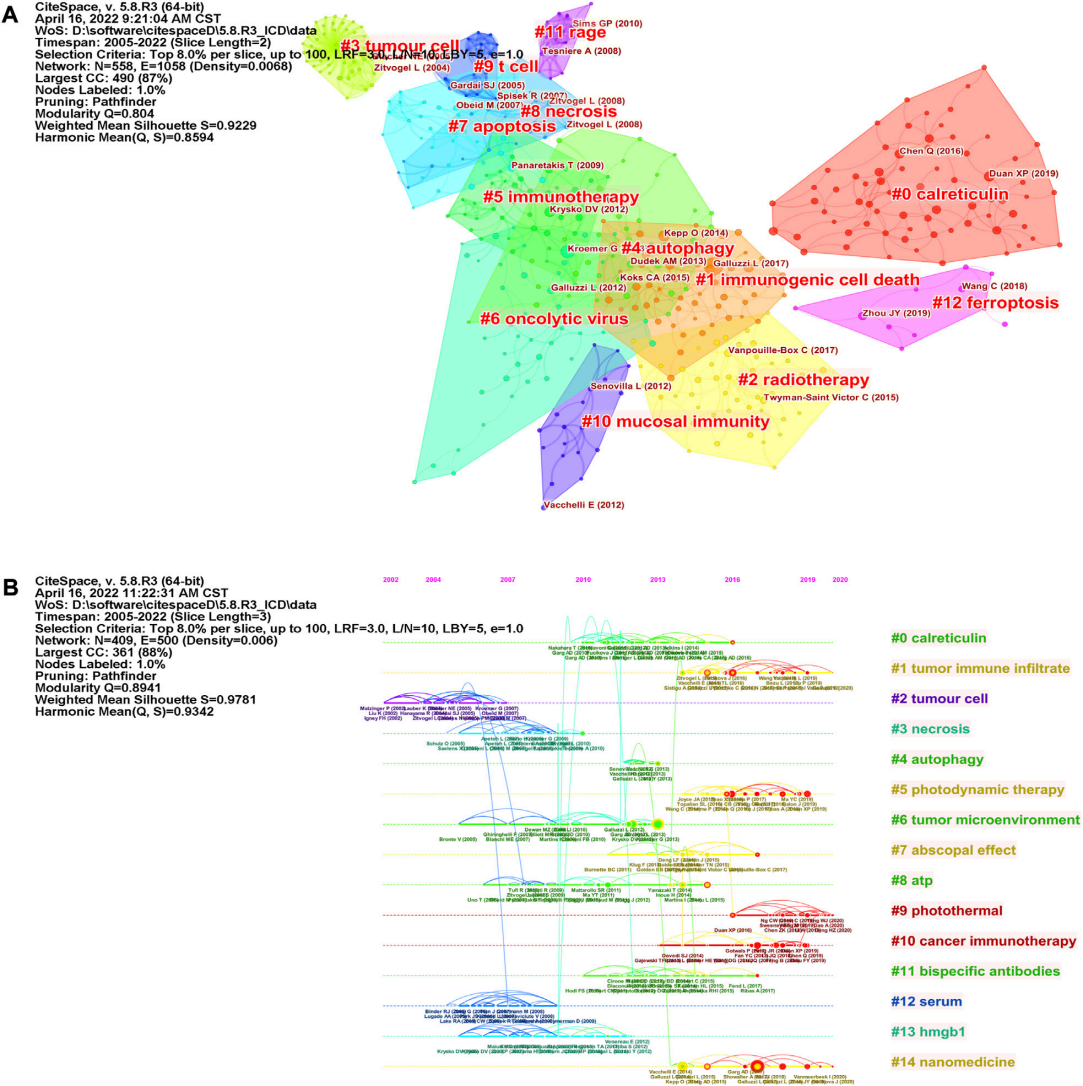
Cluster view of co-cited references using keywords as label source **(A)** and timeline view of co-cited references using title words as label source **(B)** were visualized by CiteSpace. Fifteen labeled clusters are colored on the right. The nodes on the line represented the cited references.

The co-citation network map of references was visualized by CiteSpace (Figure 6A) and 13 clusters with keywords were recognized. There were strong clustering effects and homogeneous networks (Modularity Q = 0.804, Weighted Mean Silhouette = 0.9229). We found references related to several cell death forms (“autophagy”, “apoptosis”, “necrosis” and “ferroptosis”) were clustered. Most forms of cell death have been reported to be immunogenic under some conditions (Rojo-Leon et al., 2012; Aaes et al., 2016; Demuynck et al., 2021). To demonstrate how research hotspots have changed over time, we performed a timeline view of co-cited references (Figure 6B). Fifteen clusters using title words of references as label source were demonstrated, and references appeared on the timelines in different positions and colors, based on the time of publication. The recent clusters on the timeline were “#9 photothermal”, “#10 cancer immunotherapy”, “#1 tumor immune infiltrate”, “#5 photodynamic therapy”, “#7 abscopal effect” and “#14 nanomedicine”.

**FIGURE 7:**
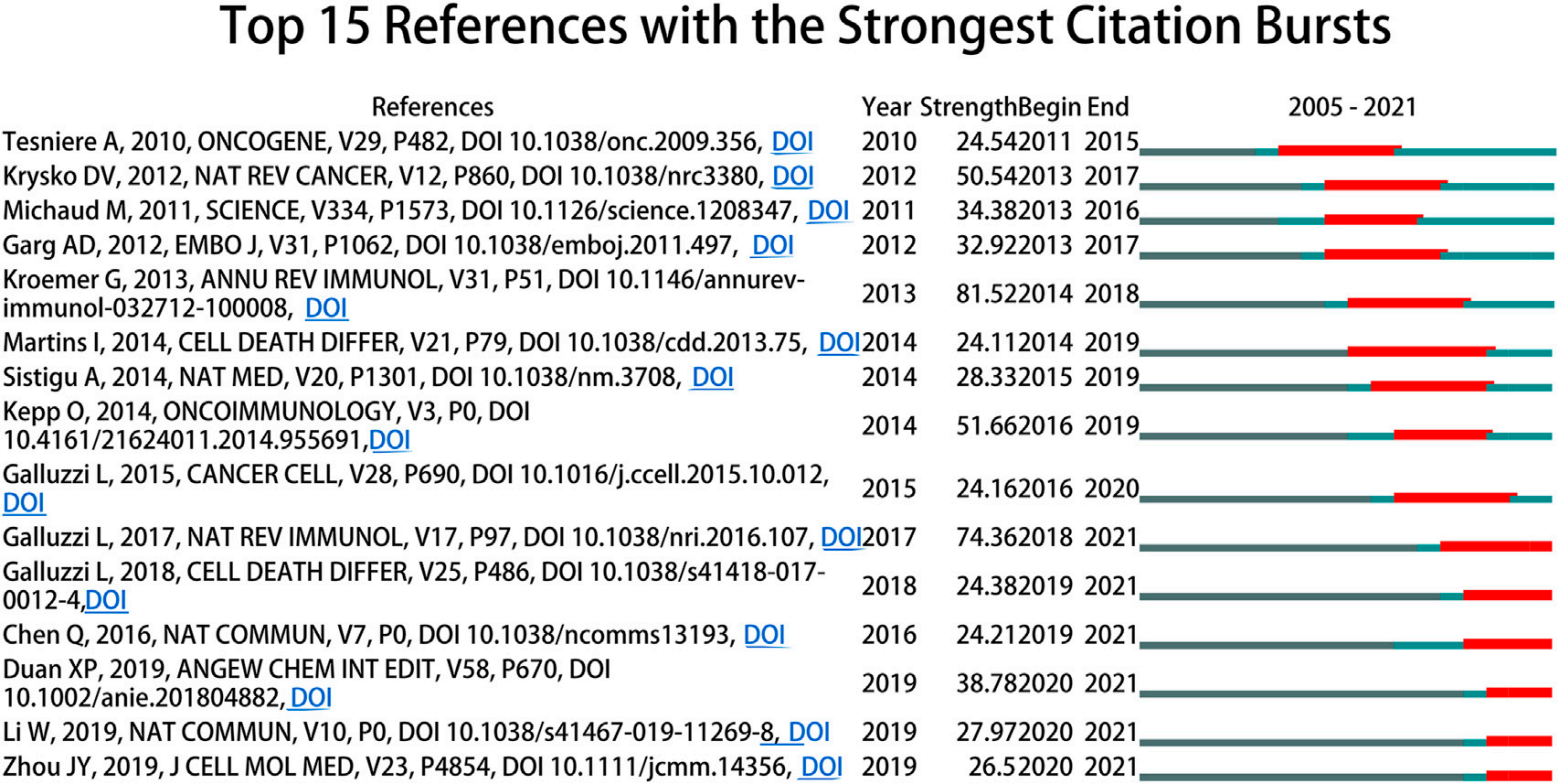
The top 15 references with the strongest citation bursts during 2005–2021.

Citation burst suggests the findings of the study have received widespread attention, which can be conducted by CiteSpace (Synnestvedt et al., 2005). Figure 7 showed that 15 references had strongest citation bursts, which were depicted by the red lines that demonstrated periods during which the references were cited most. The references that maintained their citation peaks until 2021 were “Galluzzi L, 2017, NAT REV IMMUNOL, V17, P97, DOI 10.1038/nri.2016.107” (Galluzzi et al., 2017), “Galluzzi L, 2018, CELL DEATH DIFFER, V25, P486, DOI 10.1038/s41418-017-0012-4” (Galluzzi et al., 2018), “Chen Q, 2016, NAT COMMUN, V7, P0, DOI 10.1038/ncomms13193” (Chen et al., 2016), “Duan XP, 2019, ANGEW CHEM INT EDIT, V58, P670, DOI 10.1002/anie.201804882” (Duan et al., 2019), “Li W, 2019, NAT COMMUN, V10, P0, DOI 10.1038/s41467-019-11269-8” (Li et al., 2019) and “Zhou JY, 2019, J CELL MOL MED, V23, P4854, DOI 10.1111/jcmm.14356” (Zhou et al., 2019).

### Analysis of keywords

**FIGURE 8:**
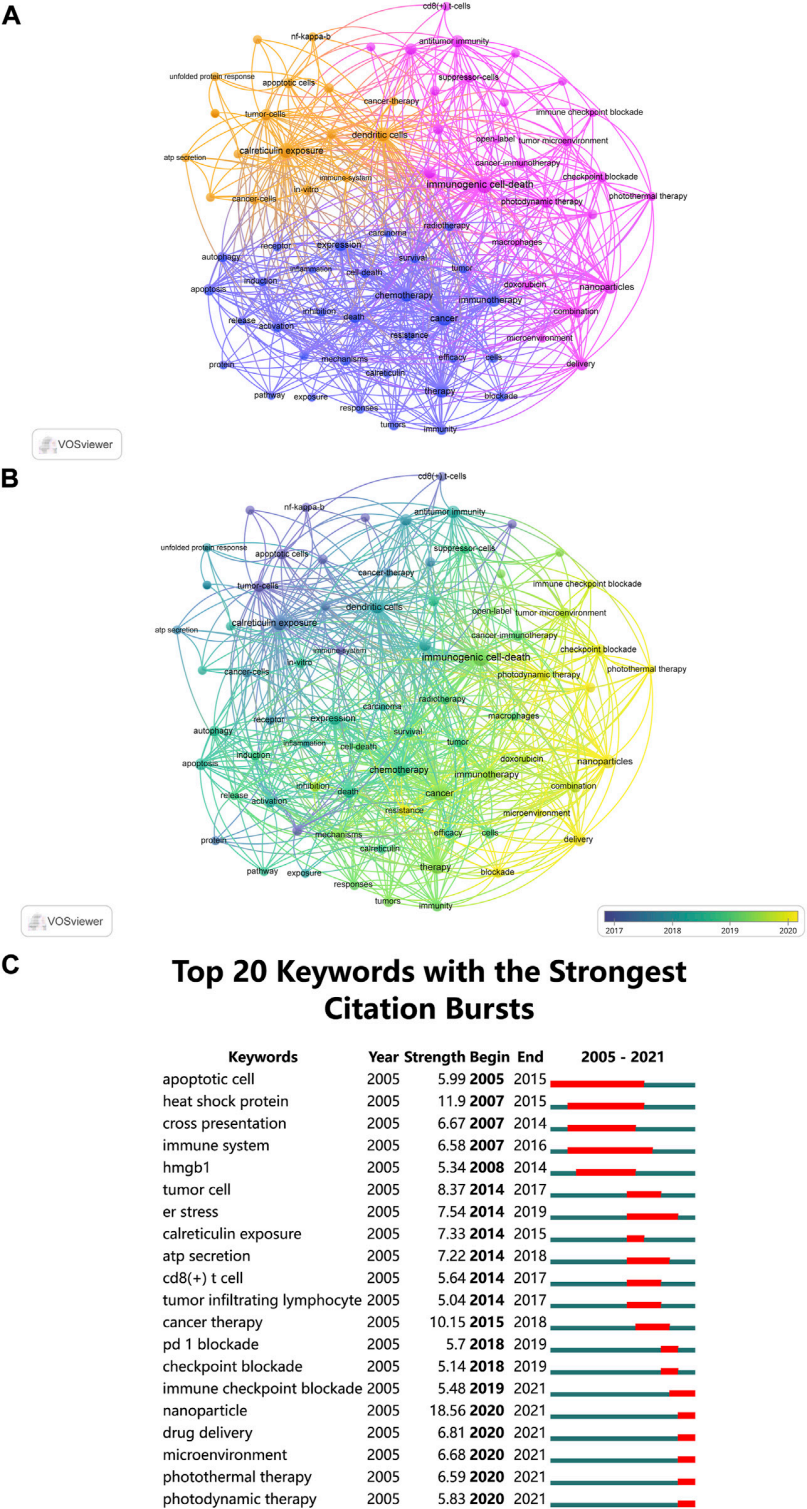
**(A)** The co-occurrence network map of author keywords, and all author keywords could be grouped into three clusters. **(B)** The overlay visualization map showed that author keywords are colored according to their average occurrence time. **(C)** Top 20 keywords (author keywords and KeyWords Plus) with the strong citation bursts.

The co-occurrence network map and overlay map of author keywords were created with VOSviewer. According to Figure 8A, author keywords with largest TLS were grouped into three clusters. In the orange cluster, researchers identified several biomarkers of ICD, including “ATP secretion” and “calreticulin exposure”, which acted as “find me” and “eat me” signals for DCs and macrophages. Using physical, nanomaterial, and other methods to induce ICD in cancer treatment was the focus of the purple cluster. The blue cluster, with author keywords of “chemotherapy”, “immunotherapy”, and “efficacy”, laid particular emphasis on the efficacy of ICD in combination with other therapies (e.g., chemotherapy and immunotherapy).

Figure 8B shows author keywords colored based on the average year they appeared. In recent years, “nanoparticles”, “delivery”, “photothermal therapy”, “photodynamic therapy” and “checkpoint blockade” appeared frequently, denoting the use of nanomaterials for drug delivery to tumor site in conjunction with photophysical approaches to induce ICD is a current research focus.

Research hotspots can also be identified by determining all keywords (author keywords and *KeyWords Plus*) with strong citation bursts (Figure 8C). The citation bursts for keywords like “immune checkpoint blockade”, “nanoparticle”, “drug delivery”, “microenvironment”, “photothermal therapy” and “photodynamic therapy” are still occurring, indicating that these fields may become new research hotspots.

## Discussion

From 2005 to 2021, the number of annual publications on ICD in cancer research steadily increased and reached a peak, indicating that it remains a research hotspot in the foreseeable future. Here, we present a bibliometric analysis that provides a comprehensive overview of the development of ICD research and predicts the future research hotspots.

Globally, the dominant countries are China, the United States and France, and they will be in a leading position in the study of ICD for many years to come. According to our analysis of institutions, six of the top 10 productive institutions were from France (Table 2), although France only ranked third in terms of publications, which may be explained by the frequent collaboration among them (Figure 3A).

When it comes to author analysis, Kroemer G, Zitvogel L, Kepp O, Garg AD and Galluzzi L were in both the top 10 most productive authors and top 10 most co-cited authors lists (Table 3). It should be noted that many of the most influential authors collaborated closely together (Figure 4A). The cited paper “Immunogenic cell death in cancer therapy” (Kroemer et al., 2013) authored by Kroemer G, Galluzzi L, Kepp O and Zitvogel L, mainly focused on the importance of triggering ICD in tumor cells for activating the immune system against cancer, as well as looked at possible factors for ICD induction failures. Galluzzi L team’s work also involved the definition of this RCD form, the development of detection methods for ICD, and the design of further treatment strategies (Kroemer et al., 2013). Therefore, we can conclude that Galluzzi L, Kroemer G, Zitvogel L et al. dominated in this field.

In terms of top journals, *Oncoimmunology, Cancers,* and *Frontiers in Immunology* can be viewed as the core journals of ICD publication, which can also guide manuscripts submission (Table 4). We also found that almost half of the top 10 journals were in the materials field, suggesting that chemotherapy, immunotherapy and other therapies combined with materials (e.g., nanoparticles) with targeting and some other physical properties are currently popular directions for triggering ICD in cancer.

Citation burst refers to that terms (e.g., references and keywords) received widespread attention and reflected dynamic changes within a certain period. In Figure 7, the active citation periods of 15 references with strongest citation bursts were relatively short, with the longest being 6 years. “Galluzzi L, 2017, NAT REV IMMUNOL, V17, P97, DOI 10.1038/nri.2016.107” (Galluzzi et al., 2017) reviewed the mechanisms that activate the immune response against dying cells. “Galluzzi L, 2018, CELL DEATH DIFFER, V25, P486, DOI 10.1038/s41418-017-0012-4” (Galluzzi et al., 2018) updated the classification of cell death subroutines, and provided molecularly oriented definitions of cell death forms interpretated by Nomenclature Committee on Cell Death (NCCD). “Chen Q, 2016, NAT COMMUN, V7, P0, DOI 10.1038/ncomms13193” (Chen et al., 2016), “Duan XP 2019, ANGEW CHEM INT EDIT, V58, P670, DOI 10.1002/anie.201804882” (Duan et al., 2019) and “Li W, 2019, NAT COMMUN, V10, P0, DOI 10.1038/s41467-019-11269-8” (Li et al., 2019) mainly focused on nanoparticle-based immunotherapy techniques, including ICIs, photodynamic and photothermal methods, that were capable of inducing and enhancing ICD in tumor cells. The main focus of “Zhou JY, 2019, J CELL MOL MED, V23, P4854, DOI 10.1111/jcmm.14356” was summarizing the emerging methods for inducing ICD in tumor cells (Zhou et al., 2019).

In bibliometrics, keywords are used to understand how a field has developed. From Figure 8B (keywords colored based on the average year they appeared) and Figure 8C (keywords with citation bursts), it is possible to see how ICD research has evolved over time.

Initially, researchers divided cell death into two categories, apoptosis and necrosis, based on cell morphology (Galluzzi et al., 2015a). And apoptosis is considered non-immunogenic (Galluzzi et al., 2015a). In later studies, apoptosis has been shown to trigger an antigen-specific immune response (Scheffer et al., 2003; Casares et al., 2005). Subsequent methods for determining whether cell death is immunogenic no longer involve morphological or biochemical assessment of dying cells, but rely on whether injection of dying cells into immunocompetent syngeneic mice can produce an immune response (Tesniere et al., 2010; Kepp et al., 2014). As tumors arise and progress, antigenic epitopes encoded by mutated genes of cancer cells have the potential to initiate immune responses. However, only neo-epitopes are usually not sufficient, adjuvants (conferred by specific DAMPs) are another indispensable induction factor for an effective anti-tumor immune response (van Kempen et al., 2015). Subsequently, several of ICD-related DAMPs were discovered, although the underlying mechanisms have not been fully elucidated until now. Obeid M et al. reported that CRT exposure determines the immunogenicity of dying cancer cells (Obeid et al., 2007a). Extracellular nucleotides including ATP released by dying cells recruit myeloid cells through P2RY2 and lead to phagocytic clearance (Elliott et al., 2009). HSP70 and HMGB1 is associated with the immunogenicity of 56°C and UVC-treated prostate cancer cells (Brusa et al., 2009). In subsequent studies, it could be observed that ICD inducers can trigger stress responses, such as autophagy and ERS, leading to the release of DAMPs required for ICD. Michaud M et al. first proposed a notion that autophagy is strictly required for releasing sufficient ATP when mouse cancer cells succumb to immunogenic chemotherapeutics (Michaud et al., 2011). It was also found that the combination of ERS inducers (such as tunicamycin) and cisplatin can effectively induce the translocation of CRT to the plasma membrane, as well as ICD, although ERS or cisplatin alone was insufficient to induce ICD (Martins et al., 2011). These biochemical indicators correlated with ICD have been identified, however, none of them (taken alone or in combination) can predict ICD with absolute certainty, suggesting that there are unknown DAMPs that remain elusive (Kepp et al., 2014). Recent studies mainly focused on combinational treatment strategies. ICD-inducing modalities can be applied to cancer treatment in combination with chemotherapy, radiation and immunotherapy. The increasing use of local radiotherapy as an immunologic adjuvant has created an opportunity for converting malignant cells into endogenous anticancer vaccines (Golden et al., 2014). The effects of chemotherapeutic drugs (such as doxorubicin, cyclophosphamide and bortezomib) on the immune system involve multiple aspects of the immune response. In 2005, doxorubicin was reported as the first ICD inducer (Casares et al., 2005). Increased number of CD8^+^ T lymphocytes and increased serum IFN-γ levels were found in metastatic tumors of DC vaccine- and doxorubicin-treated mice (Kawano et al., 2016). Nanomaterials and PDT are currently at the forefront of research on triggering ICD of cancer (Buytaert et al., 2006; Zou et al., 2016), which will be discussed further below.

CiteSpace was used to find all keywords (author keywords and *KeyWords Plus*) with citation bursts, which also helps to reveal future trends in certain area. It could be summarized that tumor microenvironment and nanoparticles are the current research hotspots based on keywords with latest citation bursts, including “immune checkpoint blockade”, “microenvironment”, “nanoparticle”, “photothermal therapy”, “drug delivery” and “photodynamic therapy” (Figure 8C).

### Tumor microenvironment

It is the immunosuppressive nature of solid tumor microenvironment that poses one of the greatest challenges to effective immunotherapy (Sharma and Allison, 2015; Huang et al., 2017). In the course of oncogenesis and tumor progression, an increasing load of somatic mutations was accumulated, leading to the generation of neo-antigens that (at least initially) are not subjected to tolerance (Schumacher and Schreiber, 2015). ICIs have been widely used to eliminate or inhibit immunosuppressive factors and received some exciting clinical responses (Pardoll, 2012; Gedeon et al., 2020; Dixon-Douglas et al., 2022). However, they are limited by being effective only in certain subgroups of patients, since neo-antigens are not only patient-specific but also tumor-specific (Pardoll, 2012). Indeed, tumor mutational load strongly predicts the response to ICIs in cancer patients (Schumacher and Schreiber, 2015). Cancer cells can downregulate surface antigens to evade recognition by immune system, and release immunosuppressive molecules to inhibit activities of effector immune cells (Chan et al., 2019; Andersen et al., 2022).

A hallmark of ICD is the release of tumor-associated antigens, DAMPs, and pro-inflammatory cytokines. Many immunogenic factors have been discovered as DAMPs. For example, ATP acts as a chemoattractant to recruit antigen-presenting cells, and CRT serves as an “eat me” signal to those cells (Obeid et al., 2007b; Garg et al., 2012). In addition, pro-inflammatory cytokines (e.g., IL-6, TNF-α, and IL-1β) induced by ICD, can also help convert an immunosuppressive tumor microenvironment into an immunogenic one (Kono and Rock, 2008). The lethal progression of malignant cells is tightly linked to the defective processes of DAMPs emission or sensing, which considerably impairs the efficiency of multiple chemo-, radio- and immunotherapeutic strategies (Hanahan and Weinberg, 2011; Galluzzi et al., 2015b). Apart from that, some suppressed upstream adaptive mechanisms, including the unfolded protein response (UPR) and autophagy, as well as destroyed signal transduction cascades that cause cell death, also contribute to cancer cell progression (Hanahan and Weinberg, 2011). How to restore the immunogenicity of malignant cells is a prominent and clinically relevant challenge, and adjuvants offer the best hope in this regard (Galluzzi et al., 2017).

### Nanoparticles

Compared to traditional strategies, nano materials can passively or actively deliver a dose of agents (e.g., ICD inducers) to specific tissues by the enhanced permeability and retention (EPR) effect or surface modification with ligands, respectively, with minimal side effects (Irvine et al., 2015; Duan et al., 2019; Wiwatchaitawee et al., 2021). The doxorubicin-liposome-microbubble complex enhances doxorubicin-induced ICD, by promoting tumor cell apoptosis, DAMPs release, and further promoting DC maturation (Huang et al., 2018). Photothermal therapy (PTT) is a treatment strategy that uses external light source (usually near-infrared light) to irradiate light-absorbing agents accumulated in tumor sites, thus converting optical energy into heat energy to kill cancer cells (Zou et al., 2016). Similarily, PDT refers to a minimally invasive therapeutic method by irradiating a photosensitizer with specific external light (Agostinis et al., 2011). During this process, excessive ROS is generated, causing oxidative stress-based cell death and enhancing tumor immunogenicity by inducing CRT exposure. PTT and PDT have been reported to have the ability to elicit antigen-specific immune responses and potentiate current immunotherapies (Marrache et al., 2013; Yata et al., 2017). Ni J et al. developed a versatile cancer cell membrane coated calcium carbonate nanoparticles which encapsulate doxorubicin (triggering ICD) and chlorine e6 (a commonly used photosensitizer), that can effectively enhance DC cell recruitment and trigger the following immune response cascade (Ni et al., 2020). Chen Z et al. developed a hybrid protein oxygen nanocarrier with chlorine e6 encapsulated for oxygen self-sufficient PDT, which offered a regimen of oxygen-augmented immunogenic PDT for cancer treatment (Chen et al., 2018). Together, nanoparticles are an ideal method that deliver light-absorbing agents, photosensitizers and ICD-inducing chemotherapeutic drugs to tumor sites to overcome immunosuppressive microenvironment (Li et al., 2019).

### Strengths and limitations

Traditional antitumor chemotherapeutic drugs are designed to inhibit tumor growth and proliferation, and usually have high cytotoxicity and low targeting, which are prone to multiple side effects and drug resistance (Nagata and Tanaka, 2017). More and more studies have found that long-term antitumor efficacy requires activation of the body’s adaptive immune response (Kono and Rock, 2008). Radiotherapy and certain chemotherapeutic agents can induce not only apoptosis but also ICD in tumor cells. The application of ICD inducers to cancer treatment is important for prolonging the survival of cancer patients (Pol et al., 2015). A variety of DAMPs produced by cells undergoing ICD have been shown to promote DC cell activation and antigen presentation with T cells, thereby activating the adaptive immune response. However, not all DAMPs are able to elicit an effective adaptive immune response (Galluzzi et al., 2017). The molecular mechanisms that underlie the release of DAMPs from dying cells remain to be further elucidated. Moreover, we still know little about how tumor cells inhibit the production of immune adjuvants. Anyhow, CRT exposure and the release of HMGB1, ATP and others, could be used as markers for the initial screening of ICD inducers *in vitro* (Galluzzi et al., 2017).

This is the first systematic analysis of ICD research through bibliometric method. All the literatures were evaluated by countries/regions, authors, organizations, journals, keywords and references to help academic researchers to gain insights into the advancements of ICD. It should be noted that, in addition to malignant diseases, ICDs have been reported in both infectious and non-infectious, non-malignant diseases (Kroemer et al., 2022). However, this study mainly focused on malignant tumors. The term ICD has emerged as part of the standard oncology vocabulary, facilitating the development of cancer treatments (Kroemer et al., 2022). While ICD is related to non-malignant diseases, the term has not been widely employed in these fields. Studying the roles of ICD in non-malignant diseases may give inspirations to oncology researchers.

## Conclusion

Recent studies have demonstrated that ICD-inducing modalities can be applied to cancer treatment in combination with nanotechnology and immunotherapy. Through studying previous high-quality articles, we have gained a better understanding of ICD. Based on a bibliometric analysis, our article provided an intuitive approach to investigate the research trends and hotspots concerning ICD in cancer treatment.

## Data Availability

The original contributions presented in the study are included in the article/Supplementary Material, further inquiries can be directed to the corresponding authors.
