# Supplementary material for: Bibliometric analysis of research on immunogenic cell death in cancer
Source: Front Pharmacol. 2022 Oct 6;13:1029020. doi: 10.3389/fphar.2022.1029020 (PMC9582244; doi:10.3389/fphar.2022.1029020)
Supplement: Supplementary file 1 [file DataSheet1.docx]

Search strategy:

#1 ((TI=(“cancer” OR “cancers” OR “neoplasm*” OR “neoplasia*” OR “carcinoma*” OR “adenocarcinoma*” OR “leukemia” OR “leukaemia” OR “sarcoma*” OR “lymphoma*” OR “oncology” OR “tumor” OR “tumors” OR “tumour” OR “tumours” OR “malignanc*” OR “malignant” OR “metastasis”)) OR AK=(“cancer” OR “cancers” OR “neoplasm*” OR “neoplasia*” OR “carcinoma*” OR “adenocarcinoma*” OR “leukemia” OR “leukaemia” OR “sarcoma*” OR “lymphoma*” OR “oncology” OR “tumor” OR “tumors” OR “tumour” OR “tumours” OR “malignanc*” OR “malignant” OR “metastasis”)) OR AB=(“cancer” OR “cancers” OR “neoplasm*” OR “neoplasia*” OR “carcinoma*” OR “adenocarcinoma*” OR “leukemia” OR “leukaemia” OR “sarcoma*” OR “lymphoma*” OR “oncology” OR “tumor” OR “tumors” OR “tumour” OR “tumours” OR “malignanc*” OR “malignant” OR “metastasis”)

#2 ((TI=(immunogenic NEAR/2 Death OR immunogenic NEAR/2 “necrotic cell death” OR immunogenic NEAR/2 apoptosis OR immunogenic NEAR/2 necrosis OR immunogenic NEAR/2 necroptosis OR immunogenic NEAR/2 pyroptosis OR immunogenic NEAR/2 ferroptosis OR immunogenic NEAR/2 killing OR “immunologic cell death”)) OR AK=(immunogenic NEAR/2 Death OR immunogenic NEAR/2 “necrotic cell death” OR immunogenic NEAR/2 apoptosis OR immunogenic NEAR/2 necrosis OR immunogenic NEAR/2 necroptosis OR immunogenic NEAR/2 pyroptosis OR immunogenic NEAR/2 ferroptosis OR immunogenic NEAR/2 killing OR “immunologic cell death”)) OR AB=(immunogenic NEAR/2 Death OR immunogenic NEAR/2 “necrotic cell death” OR immunogenic NEAR/2 apoptosis OR immunogenic NEAR/2 necrosis OR immunogenic NEAR/2 necroptosis OR immunogenic NEAR/2 pyroptosis OR immunogenic NEAR/2 ferroptosis OR immunogenic NEAR/2 killing OR “immunologic cell death”)

#2 and #3
